# Differential Expression Analysis of Chemoreception Genes in the Striped Flea Beetle *Phyllotreta striolata* Using a Transcriptomic Approach

**DOI:** 10.1371/journal.pone.0153067

**Published:** 2016-04-11

**Authors:** Zhongzhen Wu, Shuying Bin, Hualiang He, Zhengbing Wang, Mei Li, Jintian Lin

**Affiliations:** Institute for Management of Invasive Alien Species, 314 Yingdong teaching building, Zhongkai University of Agriculture and Engineering, Guangzhou 510225, PR China; USDA-ARS, UNITED STATES

## Abstract

Olfactory transduction is a process by which olfactory sensory neurons (OSNs) transform odor information into neuronal electrical signals. This process begins with the binding of odor molecules to receptor proteins on olfactory receptor neuron (ORN) dendrites. The major molecular components involved in olfaction include odorant-binding proteins (OBPs), chemosensory proteins (CSPs), odorant receptors (ORs), gustatory receptors (GRs), ionotropic receptors (IRs), sensory neuron membrane proteins (SNMPs) and odorant-degrading enzymes (ODEs). More importantly, as potential molecular targets, chemosensory proteins are used to identify novel attractants or repellants for environmental-friendly pest management. In this study we analyzed the transcriptome of the flea beetle, *Phyllotreta striolata* (Coleoptera, Chrysomelidae), a serious pest of Brassicaceae crops, to better understand the molecular mechanisms of olfactory recognition in this pest. The analysis of transcriptomes from the antennae and terminal abdomens of specimens of both sexes identified transcripts from several key molecular components of chemoreception including 73 ORs, 36 GRs, 49 IRs, 2 SNMPs, 32 OBPs, 8 CSPs, and four candidate odorant degrading enzymes (ODEs): 143 cytochrome P450s (CYPs), 68 esterases (ESTs), 27 glutathione S-transferases (GSTs) and 8 UDP-glycosyltransferases (UGTs). Bioinformatic analyses indicated that a large number of chemosensory genes were up-regulated in the antennae. This was consistent with a potential role in olfaction. To validate the differential abundance analyses, the expression of 19 genes encoding various ORs, CSPs, and OBPs was assessed via qRT-PCR between non-chemosensory tissue and antennae. Consistent with the bioinformatic analyses, transcripts for all of the genes in the qRT-PCR subset were elevated in antennae. These findings provide the first insights into the molecular basis of chemoreception in the striped flea beetle.

## Introduction

Insects have a sophisticated olfactory system that is crucial for their survival and reproduction; it provides information about food sources or oviposition sites, toxic compounds to be avoided, and pheromones used for communication. Insects sense volatile molecules via olfactory receptor neurons (ORNs) housed in chemosensory sensilla located primarily on the antennae (the predominant olfactory organ) and maxillary palps [1]. During olfactory transduction, the ORNs transform odor information into neuronal electrical signals. This process is initiated by the binding of odor molecules to receptor proteins on the ORN dendrites. Odor molecules bind antennal receptor proteins that belong to three large and divergent multigene families, namely odorant receptors (ORs) [2], ionotropic receptors (IRs) [3], and gustatory receptors (GRs) [4, 5]. In addition to the odor receptors, several multigene families encode proteins with crucial roles in insect olfaction. These include odorant binding proteins (OBPs) [6, 7], chemosensory proteins (CSPs) [8–10], sensory neuron membrane proteins (SNMPs) [11, 12] and odorant degrading enzymes (ODEs) [13].

Great strides have been made in the identification and characterization of the chemoreception gene families in insects mainly due to sequencing of insect genomes and more recently the availability of transcriptomes from chemosensory appendages [14–17]. More importantly, as potential molecular targets, chemosensory proteins can be used to identify novel attractants or repellants for use in environment-friendly pest management [18–21]. However, most of this information is from the model insects including *Drosophila melanogaster*, *Anopheles gambiae*, *Bombyx mori*, *Apis mellifera*, and *Tribolium castaneum* [22–25]. In the Coleoptera order, which contains a large number of agricultural insect pests, and apart from *T*. *castaneum*, chemoreception gene families have been identified only from a few forest or stored grain pests including the bark beetles, *Ips typographus* and *Dendroctonus ponderosae* [26], the emerald ash borer, *Agrilus planipennis* [27], the longhorned beetle, *Batocera horsfieldi* [28], the metallic green beetle, *Anomala corpulenta* [29, 30], the yellow mealworm beetle, *Tenebrio molitor* [31], the red turpentine beetle, *Dendroctonus valens* [32], and the recently published cabbage beetle, *Colaphellus bowringi* [33].

Flea beetles of the genus *Phyllotreta* (Chevrolat, Coleoptera, Chrysomelidae) are specialized herbivores that infest the Brassicaceae and related plant families. The striped flea beetle, *Phyllotreta striolata* (Fabricius), is one of the serious pests of Brassicaceae crops, and is widely distributed worldwide [34]. In southern China, this pest causes year-round damage primarily due to the climate, which is suited not only for their development but also for the continuous cultivation of their preferred food plants [35]. Currently, *P*. *striolata* control in China involves foliage application of chemical insecticides such as fipronil, chlorpyrifos, phoxim and imidacloprid [36–38]. But the emergence of insecticide resistance, growing concerns about environment contamination, as well as food safety issues have directed the search for novel, effective and environment-friendly strategies to control *P*. *striolata*. However, information on the molecular components that regulate olfaction in this beetle is lacking.

To better understand the molecular mechanisms behind olfactory recognition in the striped flea beetle, we sequenced the antennal and abdominal transcriptomes of both males and females using next generation sequencing. Analyses of the four transcriptomes led to the identification of multigene families involved in chemoreception in the striped flea beetle and initial information of their differential expression in the two tissues and both sexes.

## Methods

### Ethics statement

The striped flea beetle, *P*. *striolata* is not included in the List of Endangered and Protected Animals in China. Rather, it is a pest of worldwide importance. No specific permissions were required for these locations or activities. The locations sampled were not privately owned or protected in any way, and this field study did not involve endangered or protected species.

### Insect rearing and collection

Mass *P*. *striolata* were originally collected from an experimental field with crucifer crops at the Fujian Agriculture and Forestry University, Fujian in China (104°E, 38°N). Collected beetles were reared on *Brassica juncea* cv. Bau-Sin plants in the Zhongkai University of Agriculture and Engineering greenhouse maintained at 21–26°C, 60–80% RH and 95% soil humidity. Lighting in the green house depended on natural day light. Beetles were allowed to lay eggs on the leaves. Leaves with eggs were washed with water to collect large number of eggs, which were shipped in an artificial climate box for mass rearing at 25°C ± 1°C, 75% relative humidity and a 12 h: 12 h dark light cycle. Hatched larvae were reared on *Brassica juncea* cv. Bau-Sin plants. After 15 days, plants were cut and pots were covered with fine mesh until adults emerged. These adults (collected 2–3 days after eclosion, virgin beetles) were observed through a stereoscope to segregate the sexes based on sexual dimorphism of the antennae [39]. In order to prevent any plant volatiles from altering the expression of chemosensory genes [40], the beetles were then transferred to the new rearing cages for 16 h of food deprivation.

### RNA isolation and Illumina sequencing

After food deprivation, from male and female beetle adults (collected 3–4 days after eclosion), 1200 antennae and 200 terminal abdomens (cut from the last two abdominal segments, including the genitals) were dissected and immediately transferred to Eppendorf tubes immersed in liquid nitrogen. The frozen tissues were crushed and total RNA was isolated with the RNeasy Mini kit (Qiagen) according to the manufacturer’s protocol. RNA concentration and quality were assessed using standard procedures as recommended for Illumina sequencing.

RNASeq library preparation was performed using Illumina’s TruSeq RNAseq Sample Prep kit (Illumina, San Diego, CA, USA). cDNA libraries were quantified using the QuantiFluor^™^ dsDNA System (Promega, Fitchburg, USA). Size range of the final cDNA libraries was evaluated with an Agilent 2010 Bioanalyzer. cDNA libraries were amplified and sequenced using the cBot and HiSeq2500 from Illumina (paired-end, 2 × 100 bp; total, 200 cycles). The raw data (raw reads) in fastq format were first processed using in-house Perl scripts. Adaptor sequences, reads with more than 5% unknown bases and low quality sequences (reads with more than 50% of the quality values less than 5) were removed from the raw reads to obtain the clean reads. Each cDNA library was deep sequenced to yield 6 gb of clean data.The raw data were deposited in the NCBI Short Read Archive (SRA) database with BioProject accession number: SRP065113.

### *De novo* assembly and sequence annotation

*De novo* assembly of all clean data was accomplished using the Trinity platform to generate unigenes [41, 42] with min_kmer_cov set to 2 and all other parameters set at default. Unigenes were first analyzed using BLASTX and compared to protein databases in the NCBI nr, Swiss-Prot and Pfam (significant thresholds of E-value < 10^−5^). Proteins with the highest sequence similarity to the unigenes were retrieved along with their putative functional annotations.

### Identification of chemosensory genes, sequence alignment and phylogenetic analysis

TBLASTN searches (http://www.ncbi.nlm.nih.gov/BLAST) were used to identify putative *P*. *striolata* chemoreception genes using known insect sequences (downloaded in NCBI with the keywords [43]) as queries (significant thresholds of E-value < 10^−5^). All candidate chemoreception genes were in turn checked using BLASTX searches at the NCBI. The open reading frames (ORFs) of putative chemoreception genes were predicted using the ORF finder (http://www.ncbi.nlm.nih.gov/gorf/gorf.html) and verified by comparing the predicted sequences to the NCBI nr protein database using BLASTP as the computational tool. The signal peptides of OBPs and CSPs were predicted using SignalP 4.1 (http://www.cbs.dtu.dk/services/SignalP/) [44]. The TMDs (Trans-Membrane Domains) of ORs, IRs, GRs, and SNMPs were predicted using TMHMM 2.0 (http://www.cbs.dtu.dk/services/TMHMM) [45]. For comparative purposes, the analyzed sequences were aligned using the E-INS-I strategy in MAFFT [46] and visualized with Jalview 2.0.1 [47].

Phylogenetic analyses of *P*. *striolata* chemoreception genes were performed in conjunction with other insect chemoreception sequences in previously published data (S1 File). Amino acid sequences (after subtraction of the signal peptides in OBP and CSP datasets) were aligned using MAFFT [46]. The maximum-likelihood trees of OR GR, OBP, CSP, and SNMP were constructed using MEGA6 [48] with the corresponding best substitution model, and IR was constructed using FastTree 2.1.7 [49]. Robustness of the branches was assessed with the bootstrap method based on 1000 iterations. To ensure greater accuracy in the analyses and that the analyzed transcripts corresponded to individual genes, incomplete transcripts without sufficient overlap in alignments and transcripts less than 200 amino acids in length (apart from the OBPs and CSPs where full-length transcripts are generally shorter than 200 amino acids) were excluded from phylogenetic analyses. The phylogenetic tree was visualized in FigTree [50].

### Sex- and tissue-specific expression profiles

Clean reads were mapped back onto the assembled transcriptome and read count for each gene was obtained from the mapping results. For each sample, gene expression levels were estimated by RSEM with default parameters in Bowtie2 [51]. Expression levels were assessed in terms of FPKM values (fragments mapped per kilobase per million reads), which were calculated based on the number of mapped transcript fragments corrected for transcript length and sequencing depth [52].

Prior to DEG analysis between the four transcriptomes, the read counts were adjusted by edgeR through one scaling normalized factor. Differential expression analysis of two samples was performed using the DEGseq R package [53]. *P* value was adjusted using q value [54]. q value < 0.005&|log2(foldchange)|>1 was set as the threshold for significant differential expression. Then, the following paired-comparisons were carried out: (i) female antennae vs. male antennae, (ii) female antennae vs. female terminal abdomens, (iii) male antennae vs. male terminal abdomens, and (iv) female terminal abdomens vs. male terminal abdomens. Processing of differential expression was performed in accordance with strict criteria: differences in the FPKM values between two analyzed transcriptomes > 3-fold, in combination with a significant Bonferroni-corrected *P*-value at < 2.2 × 10^−4^. Expression levels of the chemoreception genes in the four transcriptomes are represented in a heat plot based on log-transformed FPKM values. Zero expression is represented in white.

### Quantitative real time-PCR validation

Quantitative real time-PCR (qPCR) was used to verify levels of expression of the selected OR, OBP, and CSP genes were up-regulated in the antennae in a Light Cycler 480 System (Roche Applied Science) using the SYBR Premix EX Taq (Takara, China). Total RNA isolated from the four tissues (200 male antennae, 200 female antennae, 100 male terminal abdomens, and 100 female terminal abdomens; 2–3 days after eclosion) was used to synthesize first-strand cDNA using a first-strand cDNA synthesis kit (Takara, China). Primers of the target and reference genes were designed using the Primer 3 program (http://frodo.wi.mit.edu/), and the PCR efficiency of these primers was validated before gene expression analysis. Expression levels of these genes were calculated relative to the two reference genes (*actin-1* and *GAPDH2*) using the comparative 2^−ΔΔCT^ method [55]. All primer sequences are listed in S1 Table. Negative controls without cDNA template or transcriptase were included in each experiment. Each RT-qPCR reaction was performed using three technical replicates and three biological replicates. Data analysis was performed using Prism 6.0 (GraphPad Software, CA, U.S.). Statistical significance was assessed by ANOVA followed by a Tukey multiple comparison test. A value of *P* < 0.05 was considered statistically significant.

## Results

### Overview of antennal and abdominal transcriptomes

Illumina sequencing yielded a total of 53,127,794, 52,588,754, 58,583,706, and 50,367,344 clean reads from the female antennae, male antennae, female terminal abdomens and male terminal abdomens, respectively (S2 Table). The combined Trinity assembly of the four striped flea beetle tissue transcriptomes generated 59,776 transcripts from which 43,584 non-redundant putative unigenes were predicted. A total of 15,765 unigenes were identified from the annotations against the protein databases (NCBI nr, Swis-Prot and Pfam) using the BLASTX algorithm (cut-off E-value of 10^−5^) (S3 Table). Homology search of all unigenes with other insect species showed that the highest percentage of unigenes matched to *T*. *castaneum* (48.7%), followed by *Dendroctonus ponderosae* (14.8%), *Gregarina niphandrodes* (8.6%), *Ceratitis capitata* (7.1%) and *Acyrthosiphon pisum* (1.2%). The remaining 19.5% of the sequences matched other insects (S1 Fig).

### Tissue- and sex-specificity of the chemosensory gene transcripts

**Fig 1:**
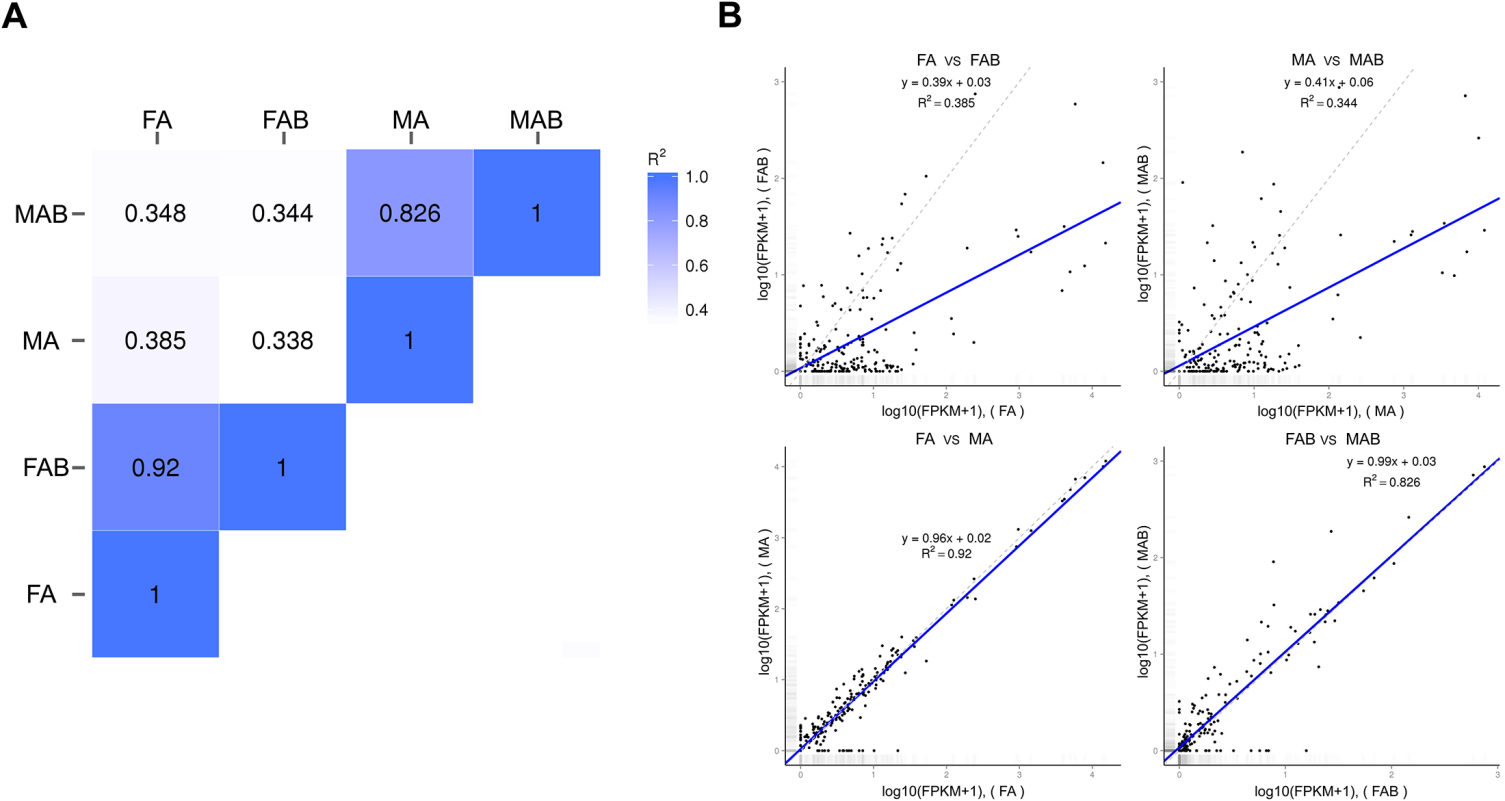
Comparison of expression profiles of the 200 chemosensory genes. A: Overall expression levels of the 200 chemosensory genes predicted from the four transcriptomes were compared using Pearson correlation coefficient (R^2^); B: Expression levels of all 200 chemoreception genes were compared pair-wise, i.e. male antennae (MA) vs. female antennae (FA); male terminal abdomens (MAB) vs. female terminal abdomens (FAB); male antennae (MA) vs. male terminal abdomens (MAB); and female antennae (FA) vs. female terminal abdomens (FAB). In addition, 1:1 plots (dotted line = 1:1 relationship) in combination with linear regression analyses (blue solid lines) indicate overall up-regulation of expression of the 200 genes in the antennae of both sexes relative to the female and male terminal abdomens. The antennae of male and female specimens expressed these genes similarly to each other and to the terminal abdomens of both sexes.

Based on homology analysis, a total of 201 chemosensory genes from six gene families (ORs, GRs, IRs, SNMPs, OBPs and CSPs), were predicted (S4–S9 Tables). Overall expression levels of the 201 chemosensory genes predicted from the four transcriptomes were compared using Pearson correlation coefficient analysis and pair-wise regression analysis (Fig 1A and 1B). Regression analysis demonstrated a close (1:1) relationship between the expression levels of the chemosensory genes in the male and female antennae (regression slope coefficient = 0.96, R^2^ = 0.92), and the overall expression level was higher in these tissues than in the terminal abdomens. Most of the chemoreception genes had the lowest expression in the female or male terminal abdomens when compared to the female or male antennae (female antennae vs. female terminal abdomens: slope = 0.39, R^2^ = 0.385; male antennae vs. male terminal abdomens: slope = 0.41, R^2^ = 0.344), respectively, although a few genes had higher expression in the terminal abdomens (see below for details). In addition, expression profiles of genes was similar in the male and female terminal abdomens (slope = 0.99, R^2^ = 0.826).

### Odorant receptors

**Fig 2:**
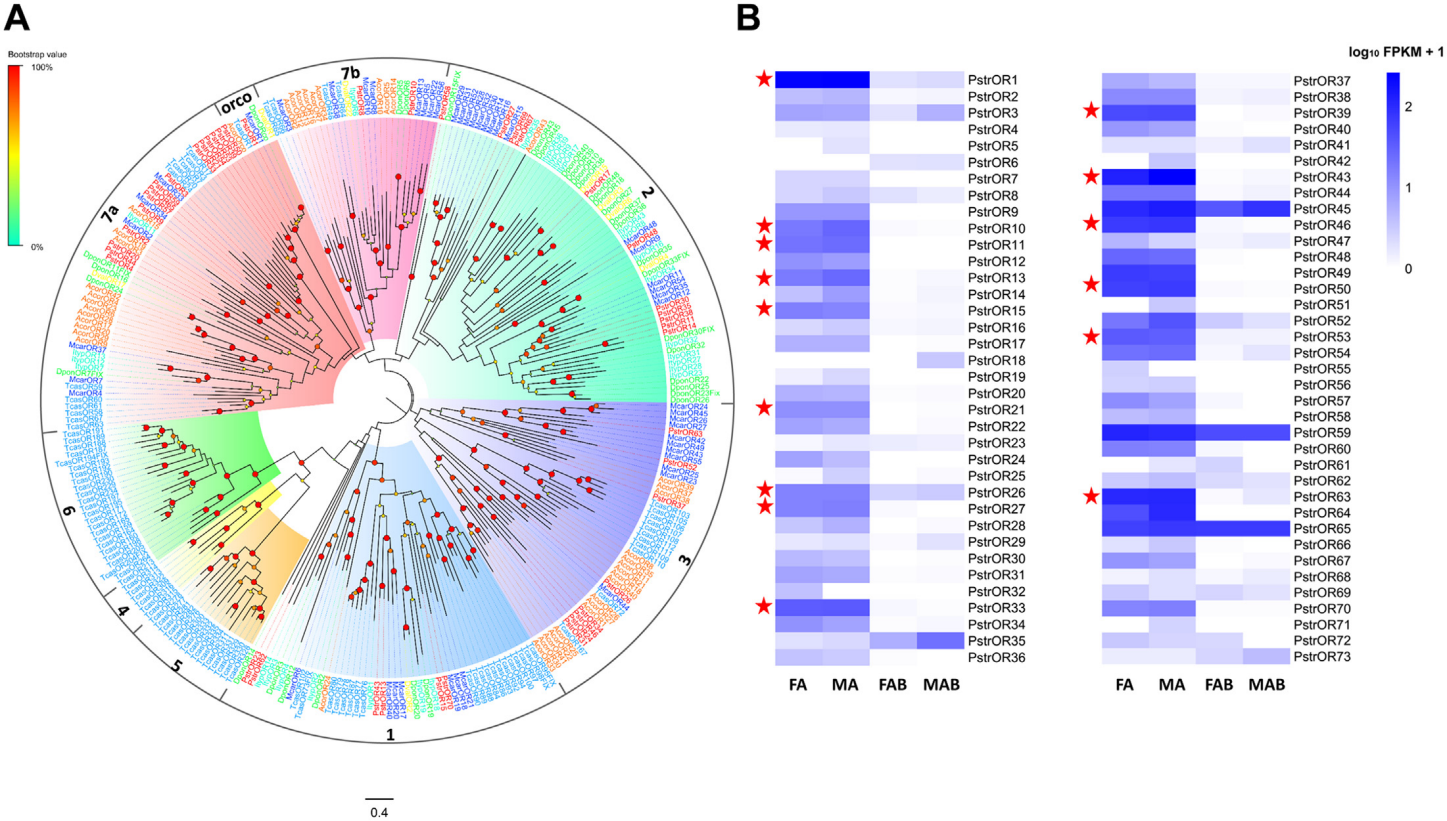
(A) Phylogenetic relationship between PstrORs and (B) their expression profiles in *P*. *striolata*.

Here, 73 OR transcripts were identified in the four transcriptome assemblies; these are referred to as PstrOR in the remainder of this article. Thirty-four of these were full-length transcripts, encoding proteins with more than 310 amino acids. The remaining were partial transcripts with low amino acid sequence identity in their overlapping regions indicating that they likely represent individual proteins. The putative PstrOR transcripts encoded complete proteins that were predicted to have three to eight transmembrane domains. Depending on the size of the partial transcripts, the remaining PstrORs were predicted to contain between zero to six transmembrane domains (S4 Table). Several OR subgroups of various sizes and structures were also distinguished. Following phylogenetic analysis, ORs from several Coleoptera species were clustered into multiple subgroups (Fig 2A) numbered from 1 to 7 according to previous studies [22, 26]. All subgroups, except for 4–6, contain a set of PstrORs proteins.

While a vast majority of the OR genes was mainly expressed in the antennae (Fig 2B; S4 Table), some members (OR35, OR45, OR59 and OR65) were also expressed in the terminal abdomens at high level. Moreover, a large proportion (15 ORs, 21%; indicated by red stars in Fig 2B; S10 Table) of the OR genes was significantly overexpressed in the antennae when compared to the terminal abdomen, but showed no differential expression between the sexes. Among them, the Orco gene (OR1) showed the highest level of expression in both sexes, followed by OR43 for which the FPKM value in the male antennae was only 15% of the FPKM value for Orco (S4 Table). In addition, some members (OR4, 5, 6, 7, 18, 19, 23, 25, 29, 32, 41, 42, 51, 55, 56, 61, 68, 71, 72, 73) with lowest expression (FPKM: 0.16–2.9; readcount less than 12) were observed in antennae or terminal abdomen of both sexes.

### Gustatory receptors

**Fig 3:**
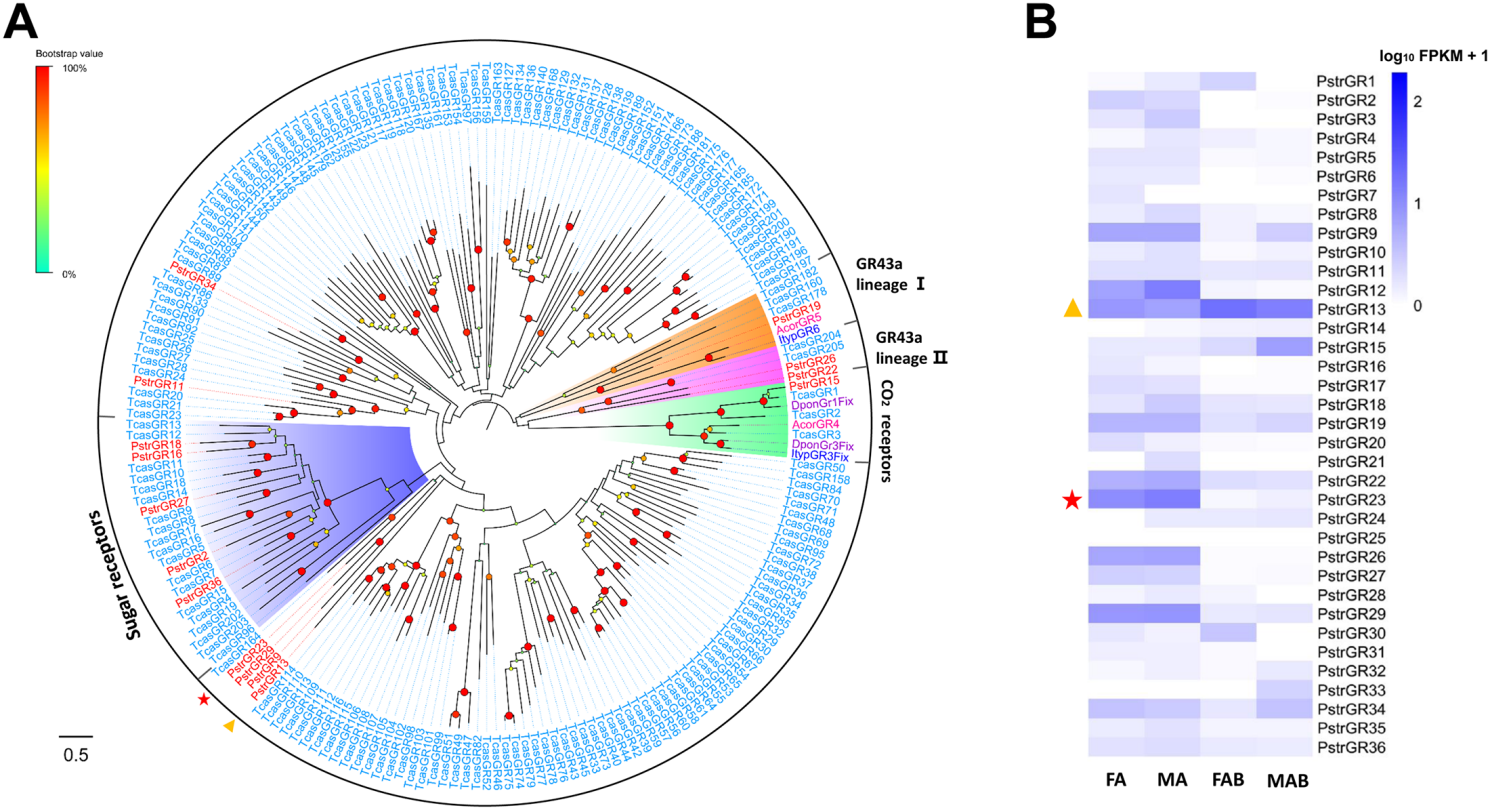
(A) Phylogenetic relationship between PstrGRs and (B) their expression profiles in *P*. *striolata*.

We identified 36 candidate GR transcripts in the four combined tissue transcriptomes (S5 Table). Thirteen of these represented full-length transcripts, encoding proteins with more than 305 amino acids. The remaining were partial fragments, encoding overlapping but distinct sequences. Consistent with other insect GRs [56], transmembrane domain and topology predictions in the full-length transcripts indicated that the most likely protein configuration had six to eight transmembrane domains with an intracellular N-terminus and extracellular C-terminus. Phylogenetic analysis revealed that only one unigene (PstrGR15) was homologous to known carbon dioxide receptors, five (PstrGR2, PstrGR16, PstrGR18, PstrGR27 and PstrGR36) were homologous to known sugar receptors, and three (PstrGR19, PstrGR22 and PstrGR26) were homologous to another known sugar receptor (GR43 lineage) (Fig 3A).

In contrast to the integral OR expression, the GR expression was low but a large proportion of these were mainly expressed in the antennae. Particularly, PstrGR23 was significantly expressed in the antennae when compared to the terminal abdomen (red start in Fig 3B), while PstrGR13 was rich in the terminal abdomen (yellow triangle in Fig 3B). Surprisingly, a lower expression of the only carbon dioxide receptor identified (PstrGR15) was found in the antennae compared with the terminal abdomen (Fig 3B).

### Ionotropic receptors

**Fig 4:**
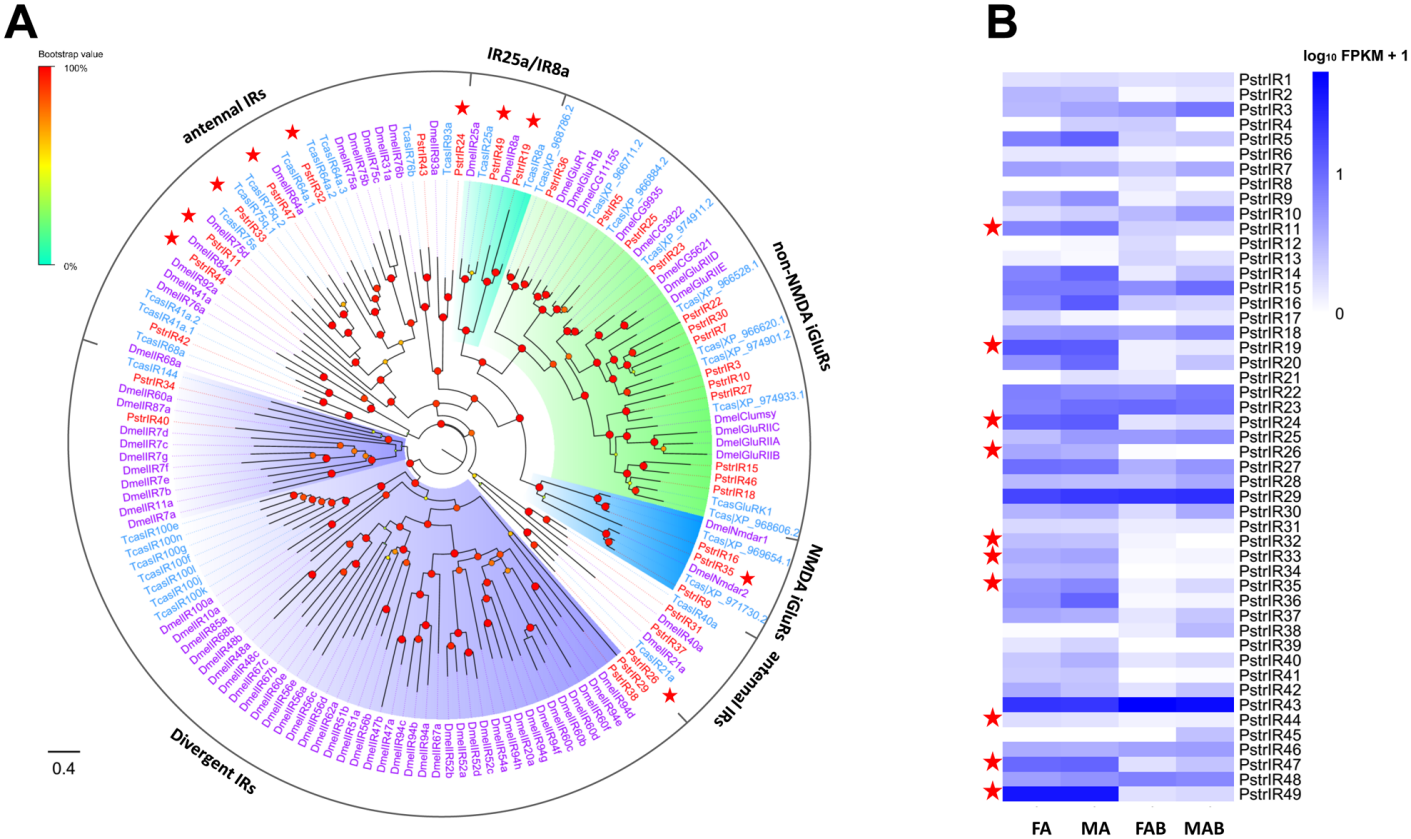
(A) Phylogenetic relationship between PstrIRs and (B) their expression profiles in *P*. *striolata*.

We identified 49 candidate IRs and iGluRs in the combined tissue transcriptomes. Twenty-three of the transcripts encoded full-length proteins with more than 339 amino acids, whereas all others represented partial transcripts. Structural and amino acid sequence alignments revealed that a majority of them shared the structural organization of insect IRs and iGluRs (in the case of co-receptors IR8a and IR25a), with the most conserved three transmembrane domains, the ligand-binding S1 and S2 domains and the ion channel pore (S2 Fig). Phylogenetic analysis demonstrated that most of the PstrIRs clustered with the IRs or iGluRs clades, only four PstrIRs (PstrIR29, PstrIR34, PstrIR38, and PstrIR40) clustered in the divergent IR clades (Fig 4A). Eleven transcripts were representative of the antennal IR groups, 13 were orthologous to non-NMDA iGluRs groups and 3 orthologous to NMDA iGluRs and 2 to IR co-receptors [3]. The sixteen PstrIRs classified as iGluRs retained all characteristic residues (R, T and D/E) [3]; the remaining IRs had diverse amino acids at one or more of these positions indicating variable ligand binding properties. Additionally, it should be noted that some of the putative PstrIRs with incomplete sequences could not be assessed for the presence of these crucial residues.

Larger numbers of IR candidates were expressed in the antennae when compared to terminal abdomen (S6 Table). Ten IR candidates were overexpressed in the antennae when compared to the terminal abdomen but showed no differential expression between sexes (Fig 4B; S10 Table). Of these, PstrIR49 (co-receptor IR25 ortholog) had the highest expression among all the IR candidates. Among the antennae-enhanced transcripts, 7 encoded antennal IRs, 2 encoded IR co-receptors and one encoded a NMDA iGluR. In the divergent IR group, all unigenes except PstrIR38 were expressed in the antennae.

### Sensory neuron membrane proteins

Two SNMP orthologs (PstrSNMP1 and PstrSNMP2) with full-length ORFs were found in the four transcriptomes (S7 Table). Phylogenetic analysis showed that PstrSNMP1 clustered with the insect SNMP1 group, and PstrSNMP2 clustered with the insect SNMP2 group (S3 Fig). The two SNMPs were both enriched in the antennae, but only PstrSNMP1 was expressed significantly more in the antennae than in the terminal abdomen. In addition, two SNMPs showed no significant difference in its expression in the female and male antennae and terminal abdomen (S10 Table).

### Odorant binding proteins

**Fig 5:**
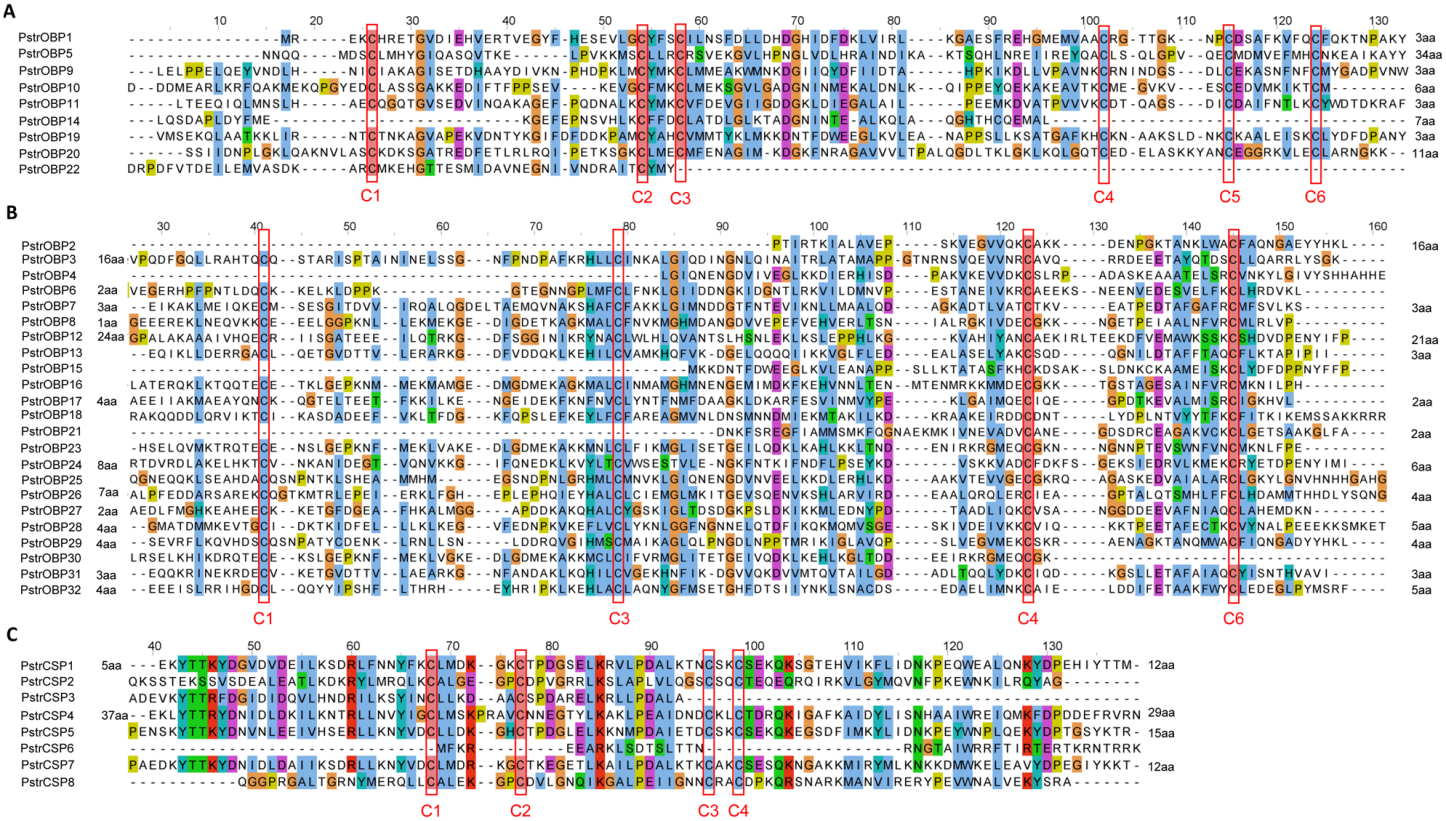
Amino acid alignment of (A) Classic OBPs, (B) Minus-C OBPs, and (C) and CSPs.

**Fig 6:**
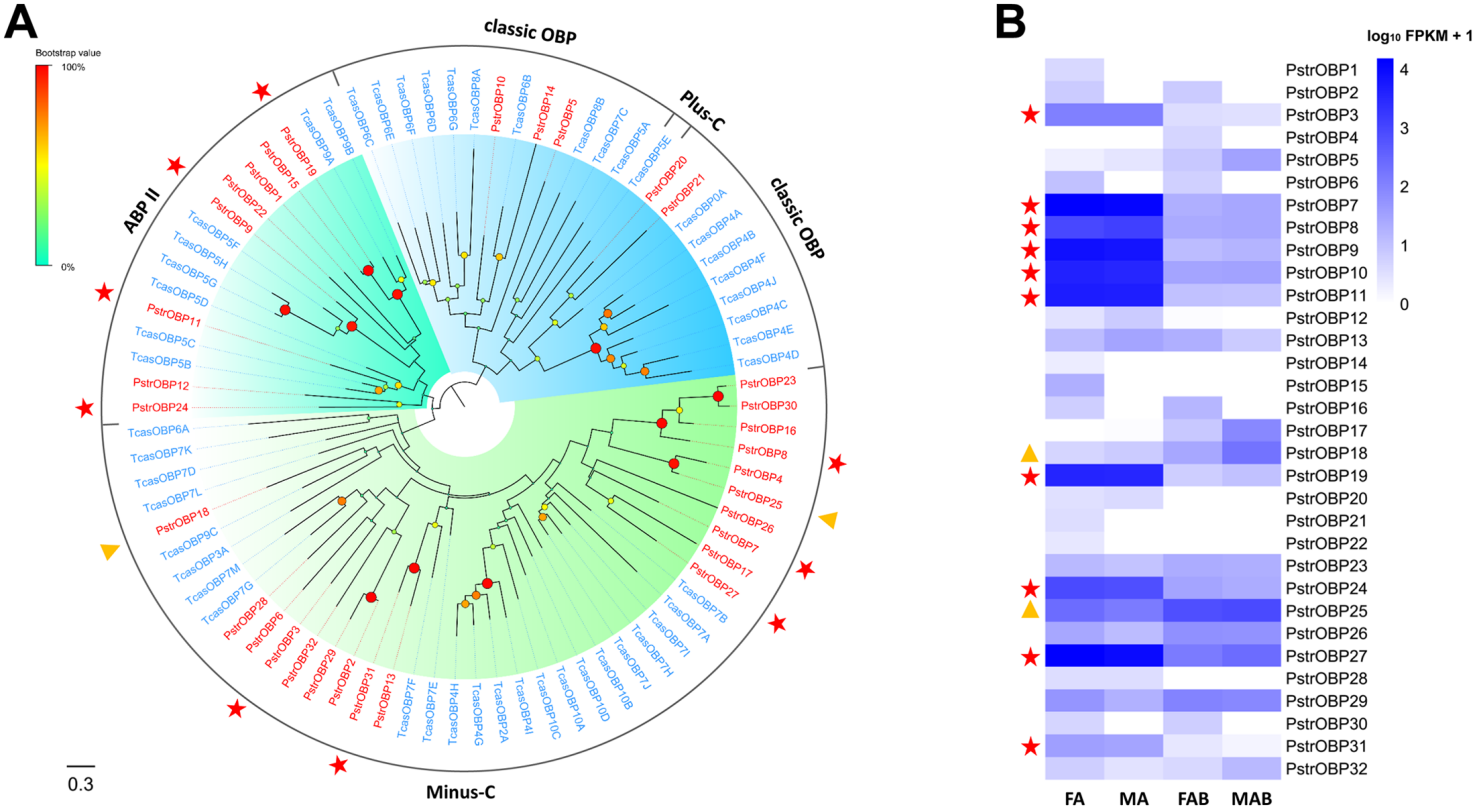
Phylogenetic relationship between PstrOBPs and their expression profiles in *P*. *striolata*.

We identified 32 OBP genes in the combined transcriptomes assembly, including 25 full-length transcripts and 7 partial ones (S8 Table). Generally, OBPs are classified into different subfamilies on the basis of cysteine residues [57]. Classic OBPs (including antennal binding proteins, ABPIIs) have six highly conserved cysteine residues forming three interlocking disulfide bonds (C_1_–C_3_, C_2_–C_5_, C_4_–C_6_) [58, 59] whereas members of the Minus-C OBP class that were derived from classic OBPs lack the C_2_–C_5_ disulfide bridge [57, 60, 61]. Among the full-length transcripts, six *P*. *striolata* OBPs (OBP1, OBP5, OBP9, OBP10, OBP11 and OBP14) were classic OBPs (Fig 5A), whereas the remaining full-length members belonged to the Minus-C OBP class (Fig 5B). In the sequence similarity dendrogram with *T*. *castaneum*, eight OBPs (OBP1, OBP9, OBP11, OBP12, OBP15, OBP19, OBP22 and OBP24) were grouped together with the ABPII sub-family from *T*. *castaneum* (Fig 6A). Members of the Minus-C OBP class, i.e. 19 PstrOBPs and 23 TcasOBPs formed a large clade. Additionally, analysis of the predicted PstrOBP amino acid sequences with signal peptide prediction algorithms suggested that all the full-length OBP, except three (OBP1, OBP3 and OBP12) had defined signal peptide sequences (S8 Table).

Five Classic OBPs and 5 Minus-C OBPs had expression in the antennae significantly higher than in the terminal abdomen (Red stars in Fig 6B; S10 Table). However, differential expression was not observed between the male and female antennae. In addition, two OBP genes (OBP12 and OBP20) were expressed exclusively in the antennae. Few OBP genes (5 classic OBP: OBP1, OBP14, OBP15, OBP21 and OBP22) were only expressed in the female antennae, while slightly lower numbers of genes (OBP2, OBP6, OBP15, OBP16 and OBP30) were expressed in both female antennae and female terminal abdomen (S10 Table). Two OBP genes (OBP18 and OBP25) showed a higher level expression (*P* < 0.05) in the terminal abdomen than in the antennae (yellow triangle in Fig 6B); OBP18 was highly expressed in the male terminal abdomens while OBP25 had higher expression in the female terminal abdomens. Although a certain number of these transcripts were present in the terminal abdomen, the overall expression level of these genes wasrelatively small.

### Chemosensory proteins

We identified 8 CSP genes in the four transcriptomes assembly (S9 Table). There were 5 full-length genes and 3 partial genes with an OS-D domain. All full-length CSPs possessed four conserved cysteine residues forming two disulfide bonds (C1–C2, C3–C4) (Fig 5C). Of these, only CSP4 deviated from the typical pattern with eight amino acid residues between C1–C2 (C1X_8_C2X_18_C3X_2_C4). The remaining followed the highly conserved pattern with four cysteines arranged with an exact spacing of C1X_6_C2X_18_C3X_2_C4. All CSPs excluding CSP5 had a predicted N terminal signal peptide with a length of 15 to 19 amino acids.

**Fig 7:**
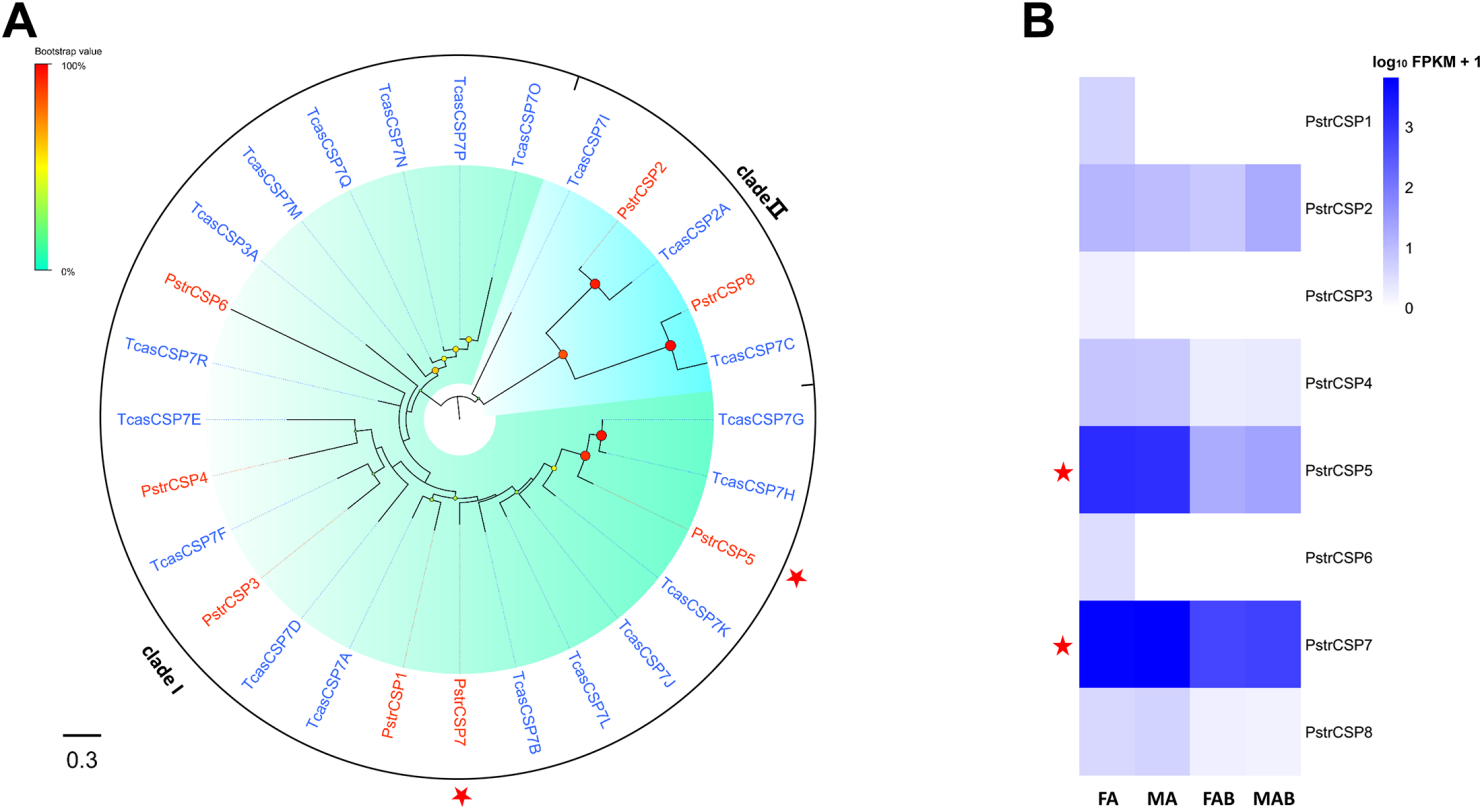
Phylogenetic relationship between PstrCSPs and their expression profiles in *P*. *striolata*.

Transcripts of only two of the eight CSPs were significantly enriched in antennae (PstrCSP5 and PstrCSP7) when compared to the terminal abdomen (S10 Table; Fig 7B). The other CSPs showed only poor expression in the tissue samples (S10 Table). In addition, phylogenetic analysis of the eight PstrCSPs showed two well distinct clades (clade I and clade II) as seen in *T*. *castaneum* CSPs with which the two PstrCSPs (CSP2 and CSP8) grouped in the “diverge” clade II. The remaining CSPs clustered with the larger group (Fig 7A).

### Odorant degrading enzymes

Odorant degrading enzymes function to rapidly inactivate signals in insect olfaction [62], and belong to detoxification enzyme classes such as cytochrome P450s (CYPs) [13, 63, 64], esterases (ESTs) [65–67], glutathione S-transferases (GSTs) [68], UDP-glycosyltransferases (UGTs) [69, 70] and aldehyde oxidases [71, 72]. A total of 43 CYPs, 68 ESTs, 27 GSTs and 8 UGTs, some of which may be involved in odorant degradation, were identified (S11 Table).

**Fig 8:**
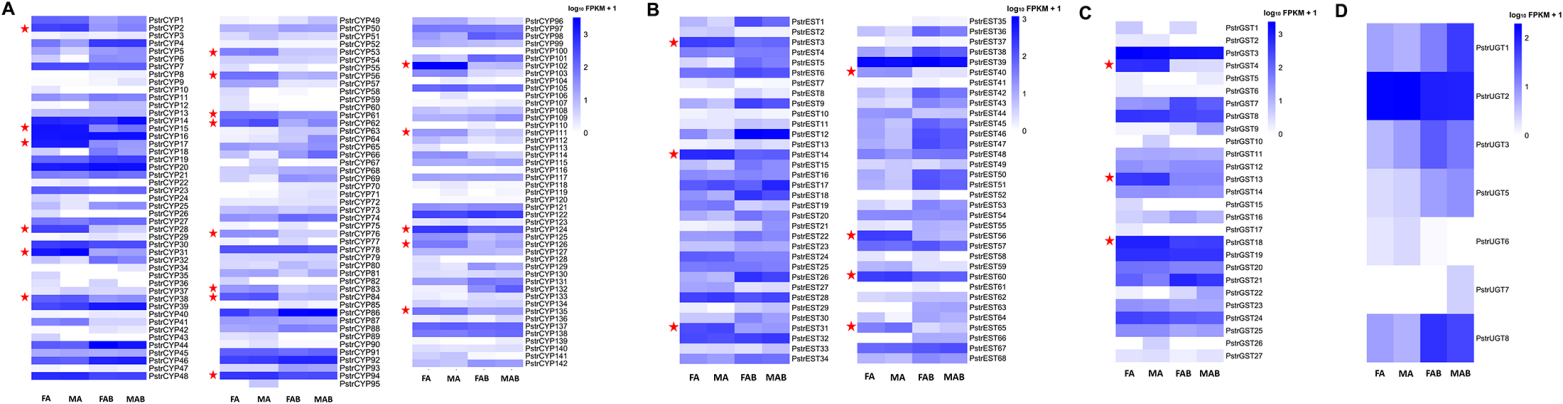
Expression profiles of (A) PstrCYPs, (B) PstrESTs, (C) PstrGSTs, and (D) and PstrUGTs.

Among the cytochrome P450s family, all candidate PstrCYPs were distributed throughout four phylogenetically distinct CYP clades, including CYP2, CYP3, CYP4, and mitochondrial clades similar to *T*. *castaneum* (S4 Fig). In addition, 19 CYPs had a higher level expression in the antennae than in the terminal abdomen (Fig 8A). Among these, six CYPs (PstrCYP31, PstrCYP53, PstrCYP56, PstrCYP62, PstrCYP126 and PstrCYP135) from the CYP4 clade been associated with the metabolism of odorants or pheromones [73]. The remaining antennal-dominant CYPs sorted with the CYP3 clade, which has been shown to be involved in xenobiotic metabolism and insecticide resistance [73]. Interestingly, some CYP4 and CYP3 P450s also clustered in a clade with a known pheromone-degrading enzyme [13] and odorant-degrading enzyme [74]. Among the other detoxification enzyme classes including ESTs and GSTs, we found 7 ESTs (PstrEST3, PstrEST14, PstrEST31, PstrEST40, PstrEST56, PstrEST60 and PstrEST65) and 3 GSTs (PstrGST4, PstrGST13 and PstrGST18) with higher expression in the antennae than the terminal abdomen (Fig 8B and 8C). In the UGTs family, none of the candidates had higher expression levels in the antennae (Fig 8D).

### qPCR validation

**Fig 9:**
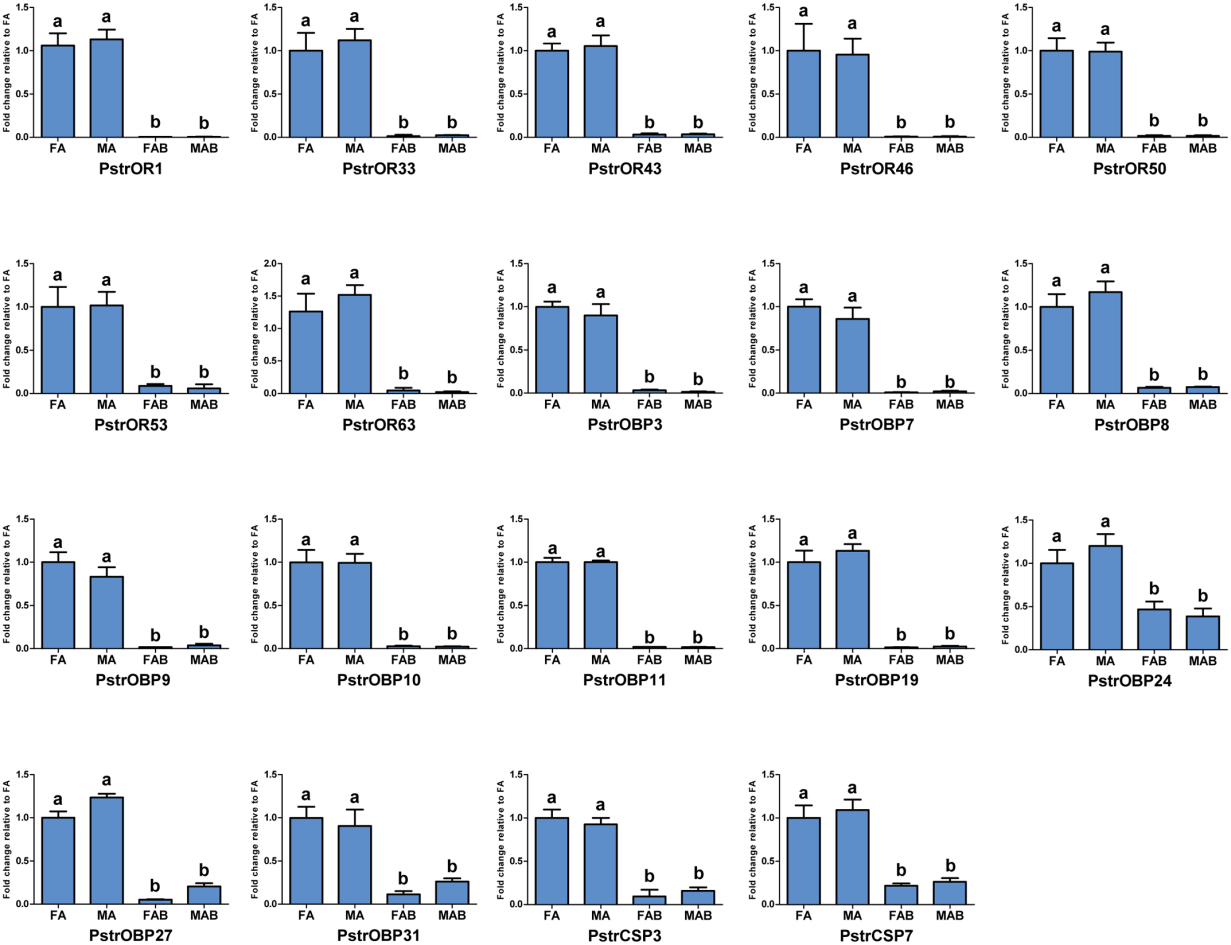
RT-qPCR results of differentially expressed genes in the antennae and terminal abdominal tissues. FA, female antennae; MA, male antennae; FAB, female terminal abdomens; MAB, male terminal abdomens. Different capital letters mean significant difference between tissues (*P* < 0.05).

To validate results of the differential abundance analyses between the antennal and abdominal transcriptomes of both male and female specimens, 19 genes encoding ORs, CSPs and OBPs that were significantly up-regulated in the antennae were selected for qPCR confirmation. The results of qPCR indicated that the selected seven PstrORs (PstrOR1, 33, 43, 46, 50, 53, 63), 10 OBPs (PstrOBP3, 7, 8, 9, 10, 11, 19, 24, 27, 31), and 2 CSPs displayed antenna-specific expression (Fig 9). These gene expression patterns were consistent with the RNA-seq data.

## Discussion

In this study, we compared the expression profiles of chemosensory genes in an olfactory and a non-olfactory tissue (terminal abdomen) in male and female *P*. *striolata* in order to identify olfaction-specific genes for use as novel targets in pest control. From the four combined transcriptomes, we identified several multigene chemoreception families, including 73 ORs, 36 GRs, 49 IRs, one SNMP, 32 OBPs, 8 CSPs, and four candidate ODE classes (143 CYPs, 68 ESTs, 27 GSTs and 8 UGTs).

Additionally, transcript abundance analysis revealed that the vast majority of chemosensory genes were up-regulated in the antennae when compared to the terminal abdomen indicating the well-established notion that chemoreception genes play a dominant role in olfaction when compared to gustatory or more general functions. Nineteen of the olfactory genes found to be significantly up-regulated in the antennae were validated by qPCR, indicating that the quality of the transcriptomic data was suitable. Our findings represent the first comprehensive analysis of the antennal and abdominal transcriptomes in *P*. *striolata* for the purpose of identifying major chemosensory gene families involved in olfaction.

### Odorant receptors

ORs are known to function as heteromeric ion channels, and consist of a highly conserved co-receptor (Orco) and a ligand-recognizing receptor (ORX) [75]. It is evident from previous studies that the known number of functional OR genes varies from 10 in the human body louse, *Pediculus humanus humanus* [76], to 350 OR genes in the fire ant, *Solenopsis invicta* [77]. These variations represent the olfaction sensing ability of insects with high level odor detection in insects harboring more odor-specific subunits (ORX). The numbers of OR-encoding transcripts identified in the *P*. *striolata* antennal and abdominal transcriptomes is much less than the number expressed only in the heads of adult *T*. *castaneum* (111 ORs), which likely reflects the narrower range of odor detection in adult *P*. *striolata*. Not surprisingly, a majority of the OR repertoire was expressed in male and female antennae, which is consistent with OR expression in other insect species such as *D*. *melanogaster* [78], *A*. *gambiae* [79], *Culex quinquefasciatus* [15], and *Mayetiola destructor* [17]. However, there was no statistical support for differences in transcript abundance between male and female antennae. It may likely be due to the homogeneous ecological interests of male and female *P*. *striolata*.

### Gustatory receptors

In this study, we found only one GR homolog of insect CO_2_ receptors with low expression in antennae. In general, insect CO_2_ receptors are highly expressed in the antennae. Since it is unlikely that CO_2_ receptor could be lost in *P*. *striolata*, we presume that an alternate organ such as the maxillary palp may be responsible for carbon dioxide detection [80, 81]. We also observed a large number of fructose receptors with high expression in the antennae. Although the exact reason for this is not clear, these receptors likely play a role in nectar feeding.

### Ionotropic receptors

A majority of the conserved antennal PstrIR genes had relatively high expression in the antennae, but showed no differential expression between the sexes. Among the antennal IR genes, we also observed some members with homology to functionally characterized genes; i.e. IR40a, which detects DEET and is a target of insect repellents [21]; IR64a, which is involved in acid detection [82]; and IR76b involved in low-salt sensing [83].

### Sensory neuron membrane proteins

SNMPs are associated with pheromone-responsive OSNs in Lepidoptera and Diptera [84, 85]. In *D*. *melanogaster*, SNMP1 is necessary for proper OSN responses to the pheromone compound, cis-vaccenyl acetate [12]. In this study, two SNMP transcripts were identified in *P*. *striolata* and classified into two sub-groups (SNMP1 and SNMP2), both of which are conserved among insect species. Of these, PstrSNMP1 was expressed significantly more in the antennae than in the terminal abdomen, but no differential expression was observed between the sexes. Expression of SNMP1 in both sexes suggests that it can detect some common components of the sex pheromone system, or alternatively, the role of SNMP1 may not be restricted to detecting sex pheromones. A similar expression pattern has also been found in other insect species [84–86].

### Odorant binding proteins and chemosensory proteins

Odorants have been known to interact with OBPs or CSPs in the sensillum lymph prior to interactions with ligand-receptors, solubilizers and carriers of odorants and pheromones. Additionally, OBP and CSP family members have been reported in other tissues and shown to be involved in non-sensory functions. OBPs and CSPs have been found in pheromone glands involved in pheromone production and release [87–93], in eggs and ovaries involved in development [94, 95], and in mouthparts involved in the dissolution of food [96, 97]. The combined transcriptomes from the four tissues in *P*. *striolata* had 32 OBPs and 8 CSPs, which are fewer than the 49 OBPs and 20 CSPs found in *T*. *castaneum* antennae and mouthparts. Notably, similar to the leaf beetle *Ambrostoma quadriimpressum* [98], no Plus-OBPs were detected in the *P*. *striolata* antennal or abdominal transcriptomes. This may indicate possible biological differences between this species and other Coleopterans [25, 26, 29, 30, 33], such as host range. Both Classic and Minus-C OBP with two cysteine motifs were found in *P*. *striolata* and other Coleopterans [25–32, 99] suggesting a common function for these OBPs in this insect order. Furthermore, in contrast to the Classic OBPs, Minus-C OBPs fewer disulfide bridges, which may increase their binding flexibility and facilitate different binding tasks [59, 100, 101]. Therefore, the antennae-specific OBPs (5 classic OBPs and 5 Minus-C OBPs) may have ‘narrow—level’ and ‘broad—level’ binding tasks. In addition, we found that the antennal expression of *P*. *striolata* ABPIIs (PstrOBP9, PstrOBP11, PstrOBP19, PstrOBP24) is similar to that of the *T*. *castaneum* homologs (Fig 6A and 6B) suggesting that the ABPII subgroup may play a specific role in olfaction. Similarly, two antennae-specific CSPs clustered with TcasCSP7G, which is highly expressed in the antennae, and TcasCSP7B, which is highly expressed in mouthparts. It is likely that these CSPs are exclusively involved in chemosensory processing. However, there was no obvious similarity in the expression pattern of Minus-C OBPs between *P*. *striolata* and *T*. *castaneum* [25].

### Odorant degrading enzymes

Multiple detoxification enzyme classes linked to odorant degradation are known in insects. These include CYPs, ESTs, GSTs, UGTs and aldehyde oxidases [102]. All except the aldehyde oxidases were identified in the transcriptomes created for this study. In addition, the number of PstrCYPs was the highest followed by PstrESTs PstrGSTs and PstrUGTs, which is consistent with the known genetic component of detoxification enzyme classes. Nineteen PstrCYPs were more highly expressed in the antennae than in the terminal abdomen, PstrCYPs s aligned with the CYP4 clade proposed to function in the degradation of pheromones while other PstrCYPs sorted to the CYP3 clade, which is linked to xenobiotic metabolism and insecticide resistance suggesting involvement of the PstrCYPs in non-chemosensory functions. Indeed, a CYP3 gene from another Coleoptera (*Dendroctonus ponderosae*) was previously characterized with odorant degrading function [74]. In addition, three other odorant degrading enzyme classes (ESTs, GSTs and UGTs) also had higher expression in the antennae than in the terminal abdomen. It is possible that multiple gene families are involved in the degradation of various odors.

## Conclusion

To better understand the molecular mechanisms regulating the olfactory recognition in the striped flea beetle, we generated antennal and abdominal transcriptomes. Differential expression analysis of chemoreception genes in the combined transcriptome assembly lead to the identification of a significant number of PstrORs, antennal PstrIRs, and ABPIIs, which are likely involved in olfaction given their predominant expression in antennae. Expression profiles of both integral and individual analyses of chemoreception transcripts (especially in ORs) revealed a lack of differential expression between the sexes suggesting homogeneous ecological interest of males and females. These findings advance our understanding of the olfactory mechanism in coleoptera and increase the gene inventory for *P*. *striolata* thus providing a valuable resource for future functional analysis of olfaction in this pest.

## Supporting Information

S1 Fig Species distribution of unigenes based on the results of the BLASTX search. (TIF)

S2 Fig Amino acid alignment of *P*. *striolata* (red) and *D*. *melanogaster* (gray) IRs and iGluRs. (TIF)

S3 Fig Phylogenetic relationship between PstrSNMP and its expression profiles in *P*. *striolata*. (TIF)

S4 Fig Phylogenetic relationship between PstrCYPs and their expression profiles in *P*. *striolata*. (TIF)

S1 File Amino acid sequences form published data in phylogenetic analyses. (DOCX)

S1 Table Primers used in qPCR analysis. (XLSX)

S2 Table Summary for the antennal and abdominal transcriptome of *P*. *striolata*. (XLSX)

S3 Table Best blast hit results obtained for search against Nr, Swiss-Prot, Pfam database and expression information in four transcriptomes. (XLSX)

S4 Table Candidate odorant receptor transcripts identified in *P*. *striolata* antennal and abdominal transcriptomes. (XLSX)

S5 Table Candidate gustatory receptor transcripts identified in *P*. *striolata* antennal and abdominal transcriptomes. (XLSX)

S6 Table Candidate ionotropic receptor transcripts identified in *P*. *striolata* antennal and abdominal transcriptome. (XLSX)

S7 Table Candidate sensory neuron membrane protein transcripts identified in *P*. *striolata* antennal and abdominal transcriptomes. (XLSX)

S8 Table Candidate odorant binding protein transcripts identified in *P*. *striolata* antennal and abdominal transcriptomes. (XLSX)

S9 Table Candidate chemosensory protein transcripts identified in *P*. *striolata* antennal and abdominal transcriptomes. (XLSX)

S10 Table Differential expressed chemoreception genes in each tissues. (XLSX)

S11 Table Candidate Cytochrome P450, esterase, glutathione S-transferase and UDP-glucuronosyltransferase transcripts identified in *P*. *striolata* antennal and abdominal transcriptomes. (XLSX)
