# Supplementary figures and images for: Differential Expression Analysis of Chemoreception Genes in the Striped Flea Beetle Phyllotreta striolata Using a Transcriptomic Approach
Source: PLoS One. 2016 Apr 11;11(4):e0153067. doi: 10.1371/journal.pone.0153067 (PMC4827873; doi:10.1371/journal.pone.0153067)

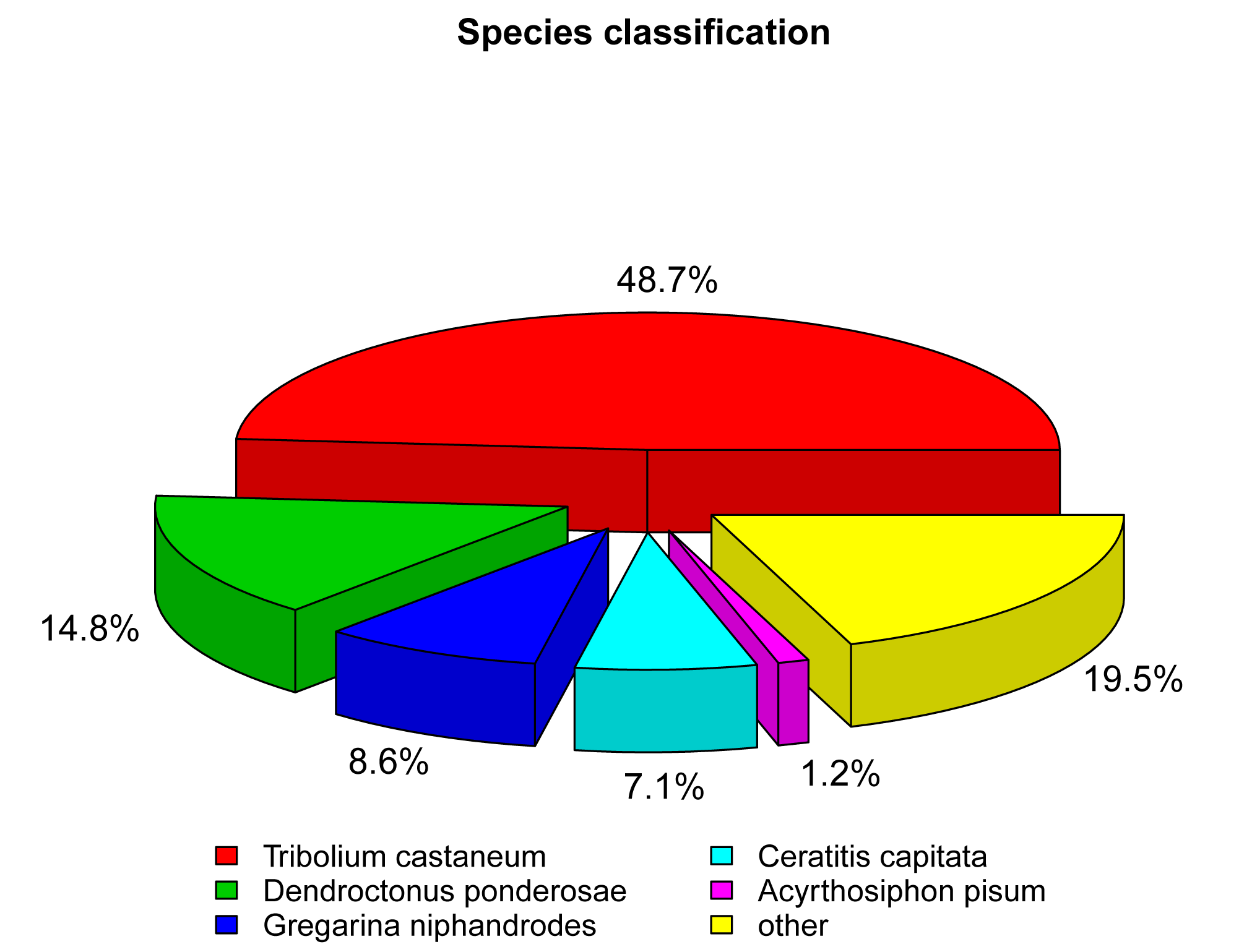

Supplement: S1 Fig — (TIF) [file pone.0153067.s001.tif]

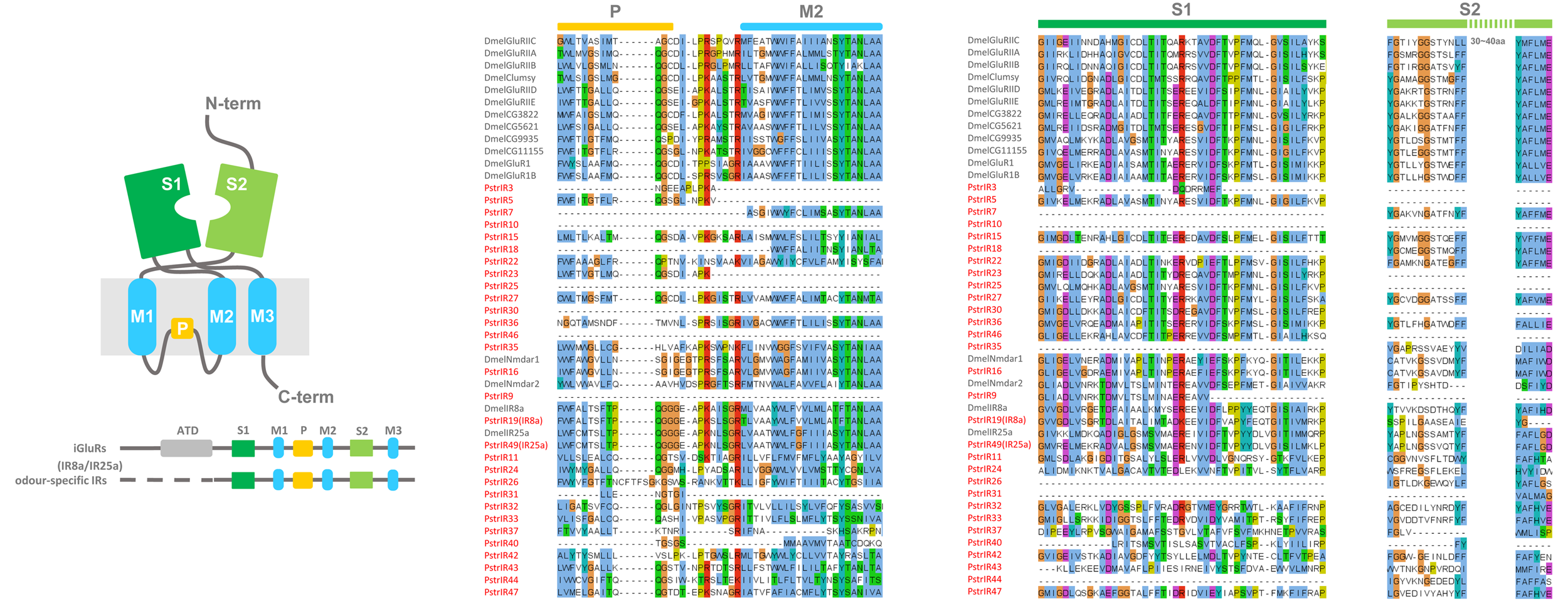

Supplement: S2 Fig — (TIF) [file pone.0153067.s002.tif]

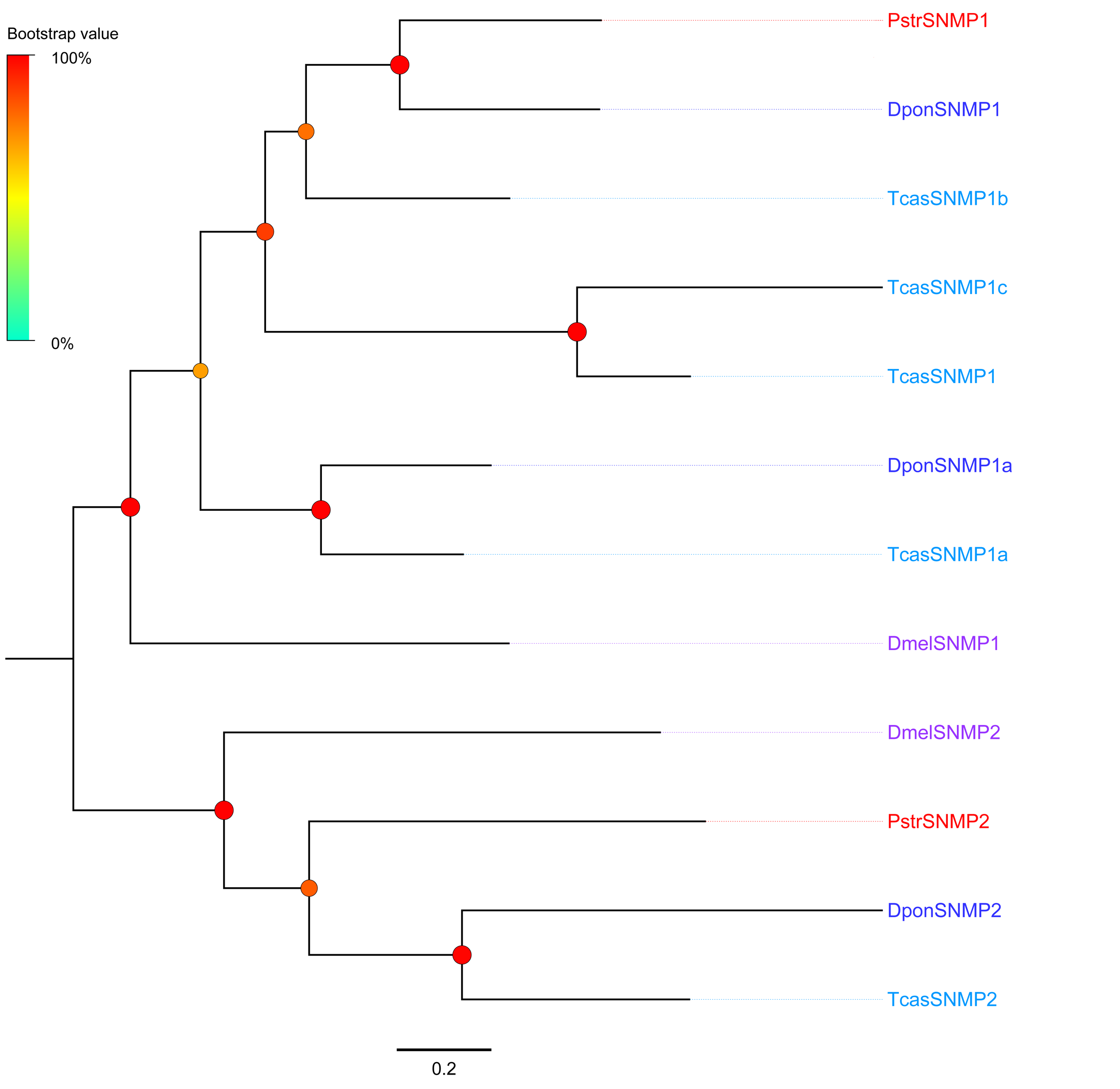

Supplement: S3 Fig — (TIF) [file pone.0153067.s003.tif]

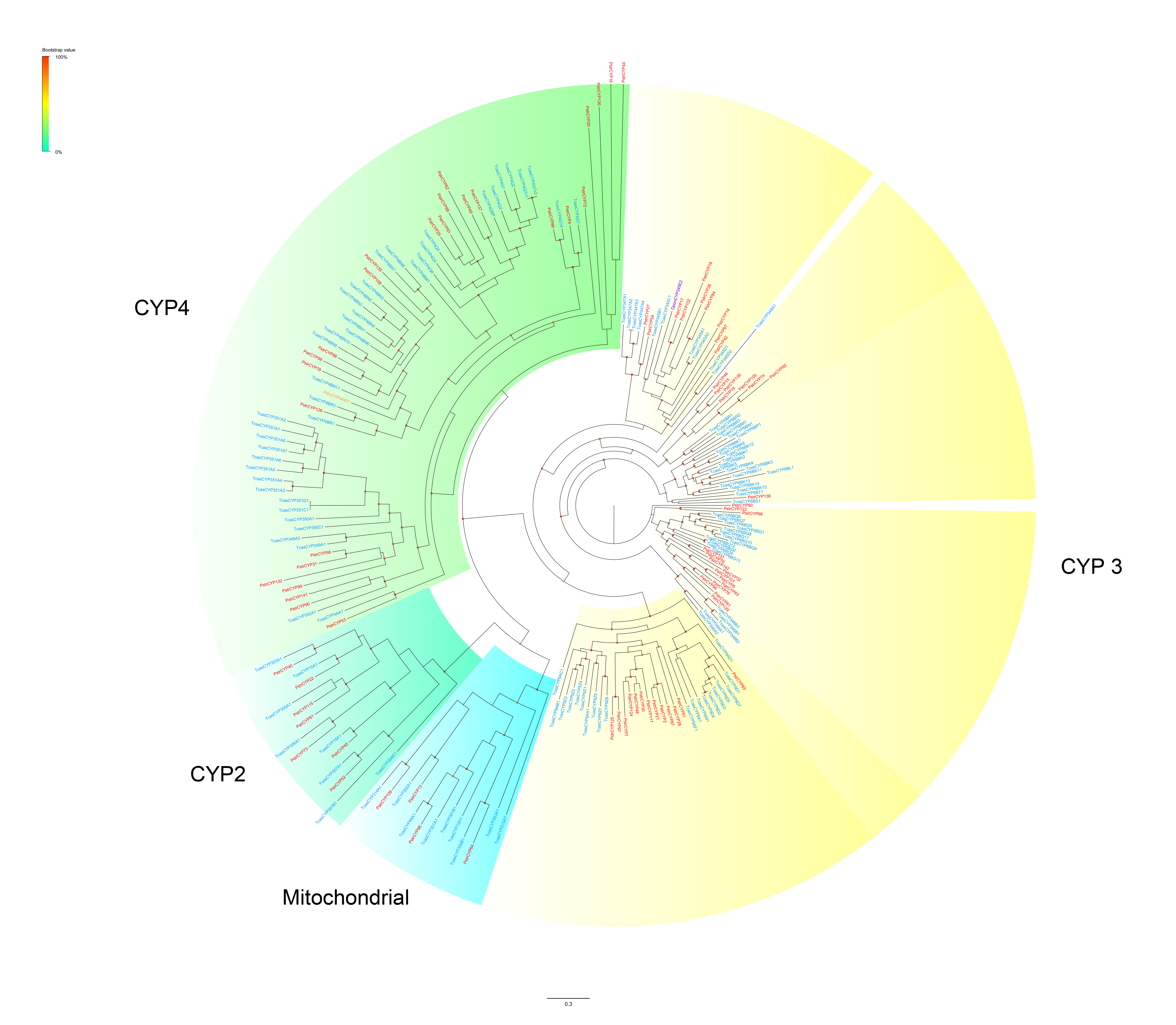

Supplement: S4 Fig — (TIF) [file pone.0153067.s004.tif]
