# Supplementary material for: Differential Expression Analysis of Chemoreception Genes in the Striped Flea Beetle Phyllotreta striolata Using a Transcriptomic Approach
Source: PLoS One. 2016 Apr 11;11(4):e0153067. doi: 10.1371/journal.pone.0153067 (PMC4827873; doi:10.1371/journal.pone.0153067)
Supplement: S1 File — (DOCX) [file pone.0153067.s005.docx]

**Amino acid sequences form published data in phylogenetic analyses**

The OR data set contained 92 protein sequences from *T. castaneum* [1], 48 from *Megacyllene caryae* [2], 41 from *Anomala corpulenta* [3], 26 from *I. typographus* and 33 from *D. ponderosae* [4], 8 from *D. valens* [5]. The GR data set contained 181 protein sequences from *T. castaneum* [6] and carbon dioxide receptors orthologous from Coleoptera [4, 7]. The IR and iGluRs data set contained 80 protein sequences from *Drosophila melanogaster* and 35 from *T. castaneum* [8]. The SNMP data set contained 7 SNMP sequences from *D. melanogaster*, *T. castaneum* [9] and 3 from *D. ponderosae* [4]. The OBP data set contained 50 protein sequences from *T. castaneum* [10]. The CSP data set contained 20 protein sequences from *T. castaneum* [10]*.*

1. Engsontia P, Sanderson AP, Cobb M, Walden KK, Robertson HM, Brown S. The red flour beetle's large nose: an expanded odorant receptor gene family in *Tribolium castaneum*. Insect Biochem Mol Biol. 2008; 38(4):387-397.
2. Mitchell RF, Hughes DT, Luetje CW, Millar JG, Soriano-Agaton F, Hanks LM, et al. Sequencing and characterizing odorant receptors of the cerambycid beetle *Megacyllene caryae*. Insect Biochem Molec. 2012; 42(7):499-505.
3. Li X, Ju Q, Jie W, Li F, Jiang X, Hu J, et al. Chemosensory Gene Families in Adult Antennae of *Anomala corpulenta* Motschulsky (Coleoptera: Scarabaeidae: Rutelinae). PloS one. 2015; 10(4):e121504.
4. Andersson MN, Grosse-Wilde E, Keeling CI, Bengtsson JM, Yuen MMS, Li M, et al. Antennal transcriptome analysis of the chemosensory gene families in the tree killing bark beetles, *Ips typographus* and *Dendroctonus ponderosae* (Coleoptera: Curculionidae: Scolytinae). BMC Genomics. 2013; 14:198.
5. Gu XC, Zhang YN, Kang K, Dong SL, Zhang LW. Antennal Transcriptome Analysis of Odorant Reception Genes in the Red Turpentine Beetle (RTB), *Dendroctonus valens*. PloS one. 2015; 10(5):e125159.
6. Richards S, Gibbs RA, Weinstock GM, Brown SJ, Denell R, Beeman RW, et al. The genome of the model beetle and pest *Tribolium castaneum*. Nature. 2008; 452(7190):949-955.
7. Chen H, Lin L, Xie M, Zhang G, Su W. De novo sequencing, assembly and characterization of antennal transcriptome of *Anomala corpulenta* Motschulsky (Coleoptera: Rutelidae). PloS one. 2014; 9(12):e114238.
8. Croset V, Cummins SF, Benton R. Ancient Protostome Origin of Chemosensory Ionotropic Glutamate Receptors and the Evolution of Insect Taste and Olfaction. J Neurogenet. 2010; 24 Suppl 1:30-31.
9. Vogt RG, Miller NE, Litvack R, Fandino RA, Sparks J, Staples J, et al. The insect SNMP gene family. Insect Biochem Molec. 2009; 39(7):448-456.
10. Vieira FG, Rozas J. Comparative Genomics of the Odorant-Binding and Chemosensory Protein Gene Families across the Arthropoda: Origin and Evolutionary History of the Chemosensory System. Genome Biol Evol. 2011; 3:476-490.
